# Complicated tracheal diverticulitis

**DOI:** 10.36416/1806-3756/e20240212

**Published:** 2024-12-20

**Authors:** Izabel de Oliveira Karam, Daniel Vaccaro Sumi, Eduardo Kaiser Ururahy Nunes Fonseca

**Affiliations:** 1. Departamento de Diagnóstico por Imagem, Hospital Israelita Albert Einstein, São Paulo (SP) Brasil.

**Figure 1:**
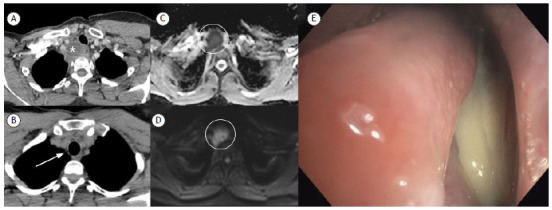
Panel A shows a hypodense mass (asterisk) in the right tracheoesophageal sulcus with mild peripheral enhancement and adjacent fat densification, suggestive of an abscess. A 9-year prior chest CT revealed an uncomplicated diverticulum at the same site (arrow in panel B). A diffusion-weighted MR image (panel C) and an apparent diffusion coefficient map (panel D) also confirmed the diagnosis of an abscess (circle in both panels). A bronchoscopy image (panel E) shows purulent secretion in the airway.

A 64-year-old female presented to the emergency department with neck pain and swelling. Further CT investigation revealed a hypodense mass in the right tracheoesophageal sulcus with mild peripheral enhancement and adjacent fat stranding (Figure 1A). The diagnosis of a tracheal diverticulum abscess was confirmed after reviewing a 9-year prior CT that revealed a tiny uncomplicated diverticulum at the same site (Figure 1B). An MRI confirmed the diagnosis showing significant restriction in diffusion-weighted images (Figures 1C and 1D). The patient then underwent echoendoscopy and bronchoscopy (Figure 1E), which revealed purulent secretion in the airway.

Tracheal diverticula are divided by their origin into congenital and acquired, both being mostly asymptomatic and incidentally found during imaging exams.^1^ When present, the most common symptoms are cough, dyspnea, stridor, dysphagia, local tenderness, and dysphonia due to local compression.^2^ Hospitalization or emergency treatment is rarely required.^3^
